# Supplementary material for: Splice-Junction-Based Mapping of Alternative Isoforms in the Human Proteome
Source: Cell Rep. Author manuscript; Available in PMC 2020 Jan 15. (PMC6961840; doi:10.1016/j.celrep.2019.11.026)
Supplement: 3 [file NIHMS1546469-supplement-3.zip › DF2/PXD009737/TestisGluC-271-Q9H0E9-IQQTPNTVAASPAASE.pdf]

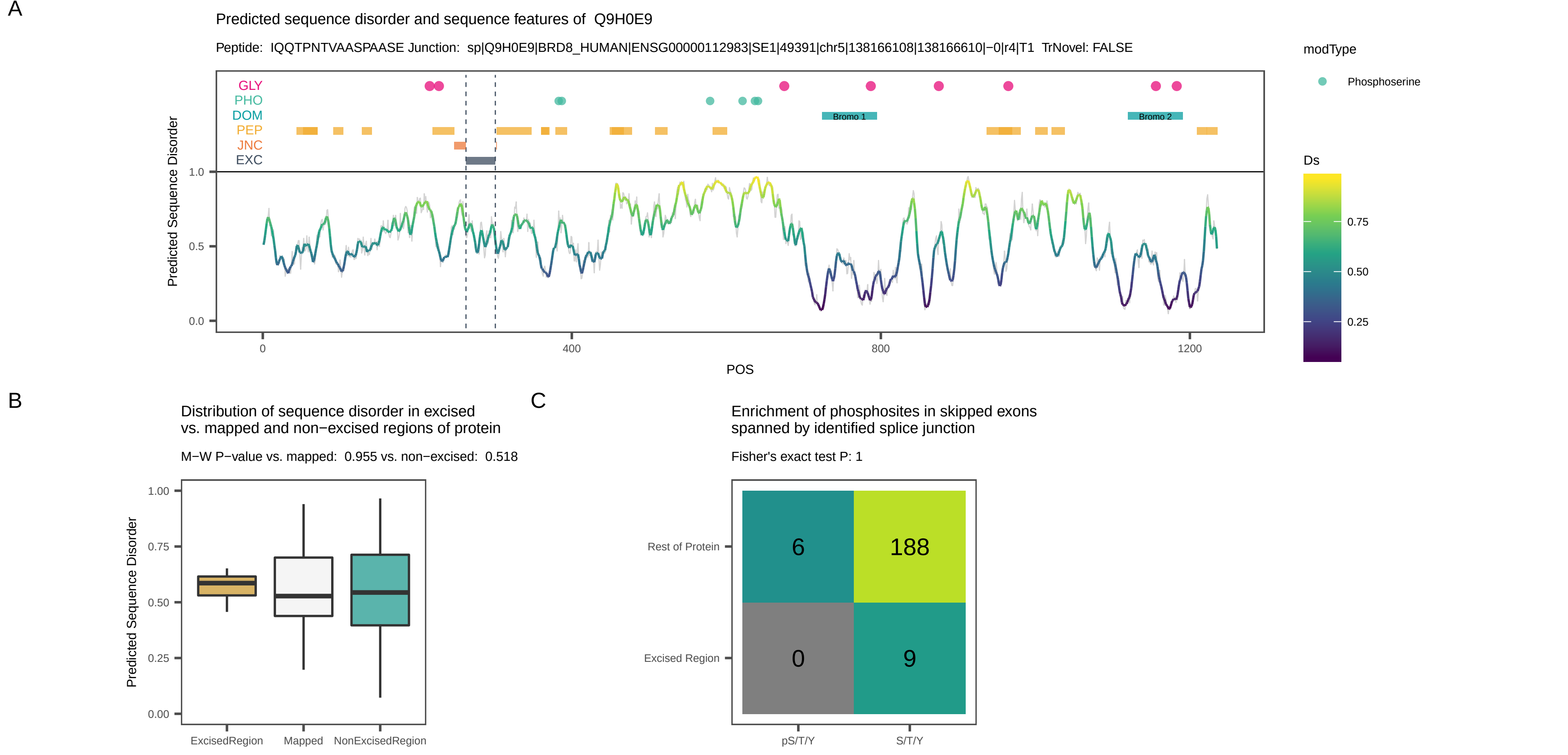
A
Predicted sequence disorder and sequence features of Q9H0E9
Peptide: IQQTPNTVAASPAASE Junction: sp|Q9H0E9|BRD8_HUMAN|ENSG00000112983|SE1|49391|chr5|138166108|138166610|−0|r4|T1 TrNovel: FALSE
GLY
PHO
DOM
PEP
JNC
EXC
Bromo 1
Bromo 2
Predicted Sequence Disorder
1.0
0.5
0.0
0
400
800
1200
POS
modType
Phosphoserine
Ds
0.75
0.50
0.25
B
Distribution of sequence disorder in excised
vs. mapped and non−excised regions of protein
M−W P−value vs. mapped: 0.955 vs. non−excised: 0.518
Predicted Sequence Disorder
1.00
0.75
0.50
0.25
0.00
ExcisedRegion
Mapped
NonExcisedRegion
C
Enrichment of phosphosites in skipped exons
spanned by identified splice junction
Fisher's exact test P: 1
Rest of Protein
Excised Region
6
188
0
9
pS/T/Y
S/T/Y
